# Superior sagittal sinus thrombosis presented as a migraine headache with normal D-dimer: a case report and literature review

**DOI:** 10.1097/MS9.0000000000005180

**Published:** 2026-05-26

**Authors:** Mohammedsadeq A. Shweliya, Khadeeja Ali Hamzah, Yousif Hameed Kurmaha, Kevin Thomas Mathew, Khalid Radwan Alsaadany, Hassan. H. Eladl, Abdulhadi M. A. Mahgoub, Yusur Amer, Sura Samer, Ali Saad Al-Shammari

**Affiliations:** a Department of Medicine, University of Baghdad College of Medicine, Baghdad, Iraq; b Department of Medicine, Al-Kindy College of Medicine, University of Baghdad, Baghdad, Iraq; c Department of Medicine, College of Medicine, University of Kufa, Najaf, Iraq; d Department of Medicine, David Tvildiani Medical University, Tbilisi, Georgia; e Department of Medicine, Mansoura University Faculty of Medicine, Mansoura, Egypt; f Department of Medicine, Faculty of Medicine Ain Shams University, Cairo, Egypt; g Faculty of Medicine, University of Gezira, Wad-Medani, Gezira, Sudan; h Department of Medicine, Mustansiriyah University, Baghdad, Iraq; i Department of Medicine, College of Medicine University of Babylon, Hilla, Iraq

**Keywords:** case report, D-dimer, migraine, superior sagittal sinus thrombosis

## Abstract

**Introduction:**

Superior sagittal sinus thrombosis (SSST) is a rare type of cerebral venous sinus thrombosis (CVST), characterized by blood clot formation in the superior sagittal sinus, leading to increased intracranial pressure. This report highlights a case of SSST presenting atypically as a migraine with normal D-dimer levels, emphasizing the need for thorough evaluation despite normal laboratory results in at-risk individuals.

**Presentation:**

A 49-year-old female experienced a severe unilateral headache, photophobia, dizziness, and neck tension. She had a history of migraines and hypertension. The patient was a heavy smoker and used combined oral contraceptives. Examination revealed neck stiffness and a blood pressure of 150/90 mmHg. Computed tomography and D-dimer tests were normal. Magnetic resonance venography (MRV) revealed SSST and a lacunar infarction. Anticoagulation was initiated, resulting in a good recovery and discharge after 11 days.

**Discussion:**

This case illustrates the difficult diagnostic challenge caused by SSST, which initially presented as a primary headache disorder. The occurrence of red flags, including an unusual headache pattern and neck stiffness, justified further investigation. The article also considers the limitations of using D-dimer as a rule-out test in high-suspicion cases, the absolute diagnostic value of MRV, and the effectiveness of standard anticoagulation therapy.

**Conclusions:**

This case serves as a reminder that clinical judgment should take precedence over normal laboratory results in patients with unusual headaches and risk factors for CVST. Patients with high-risk and persistent or unusual headaches should be immediately referred for advanced neuroimaging, such as MRV, to facilitate early diagnosis and treatment.

## Introduction

Superior sagittal sinus thrombosis (SSST) is a rare form of cerebral venous sinus thrombosis (CVST). It occurs due to the formation of a blood clot in the superior sagittal sinus, one of the major veins involved in blood drainage^[^1,2^]^. SSST can result in cerebral edema, increased intracranial pressure, and other neurological consequences due to compromised venous drainage and elevated intracranial pressure^[^3,4^]^.

HIGHLIGHTS

- Superior sagittal sinus thrombosis, presenting atypically as a migraine.
- Normal D-dimer does not rule out the need to evaluate for venous thrombosis.
- Highlights the importance of considering risk factors that increase the likelihood of thrombosis, such as smoking and the use of oral contraceptive pills.

CVST accounts for 0.5–1% of all strokes and commonly affects younger individuals, especially women of childbearing age, due to hormonal and prothrombotic risk factors^[^5^]^. Mortality rates for SSST vary based on factors such as age, intracerebral hemorrhage, and associated complications, typically ranging from 4 to 16%, depending on disease severity and time to hospital referral^[^6–8^]^.

SSST can present with a wide range of symptoms, usually symptoms of increased intracranial pressure and localized cerebral damage. The most common symptom is a persistent headache, occurring in 50–80% of the patients^[^9^]^. Other manifestations include seizures, hemiparesis, hemianesthesia, blurred vision, and a disturbed level of consciousness. In severe cases, symptoms may worsen and lead to brain herniation and coma^[^3,10^]^.

Multiple risk factors are implicated in SSST. Acquired conditions, such as oral contraceptive pills, dehydration, infections – especially in dangerous areas of the face – malignancies, and systemic inflammatory diseases like systemic lupus erythematosus and Behçet’s disease, can increase the risk of SSST^[^11–14^]^. Additionally, inherited thrombophilic conditions, such as protein C and S deficiencies, antithrombin III deficiency, hyperhomocysteinemia, and factor V Leiden mutation, also increase the risk of SSST^[^15,16^]^.

Diagnostic workup for SSST includes laboratory and radiological investigations. D-dimer represents a valuable screening tool for CVST. D-dimer levels correlate with the degree of sinus involvement, which helps in the rapid evaluation and diagnosis^[^17,18^]^. The main focus in diagnosing SSST is neuroimaging, where magnetic resonance venography (MRV) is the gold standard for diagnosis^[^19,20^]^. A common alternative, especially in the ER, is computed tomography (CT) venography, which demonstrates “empty delta” signs^[^21,22^]^.

This report describes a case of SSST with an atypical presentation as migraine and normal D-dimer levels. By highlighting the limitations of initial diagnostic tests, this case aims to reinforce the importance of maintaining a high clinical suspicion and proceeding to advanced neuroimaging when red flags are present, thereby minimizing diagnostic delays.

## Case presentation

A 49-year-old Middle Eastern woman came to the clinic complaining of a severe headache that had been present for 5 days. She explained that the pain was unilateral (right-sided), pounding, and started upon waking. Besides the headache, she also experienced photophobia, dizziness, and tension in her occipital and neck regions. Her past medical history revealed that she had been diagnosed with migraines 11 years earlier (her attacks used to last for 2–3 hours), and she also had hypertension. Currently, she was taking paracetamol and meloxicam as needed for pain relief and candesartan cilexetil for blood pressure control. She was a heavy smoker (smoking 2-3 packs per day) and was on combined oral contraceptive pills.

**Figure 1.**
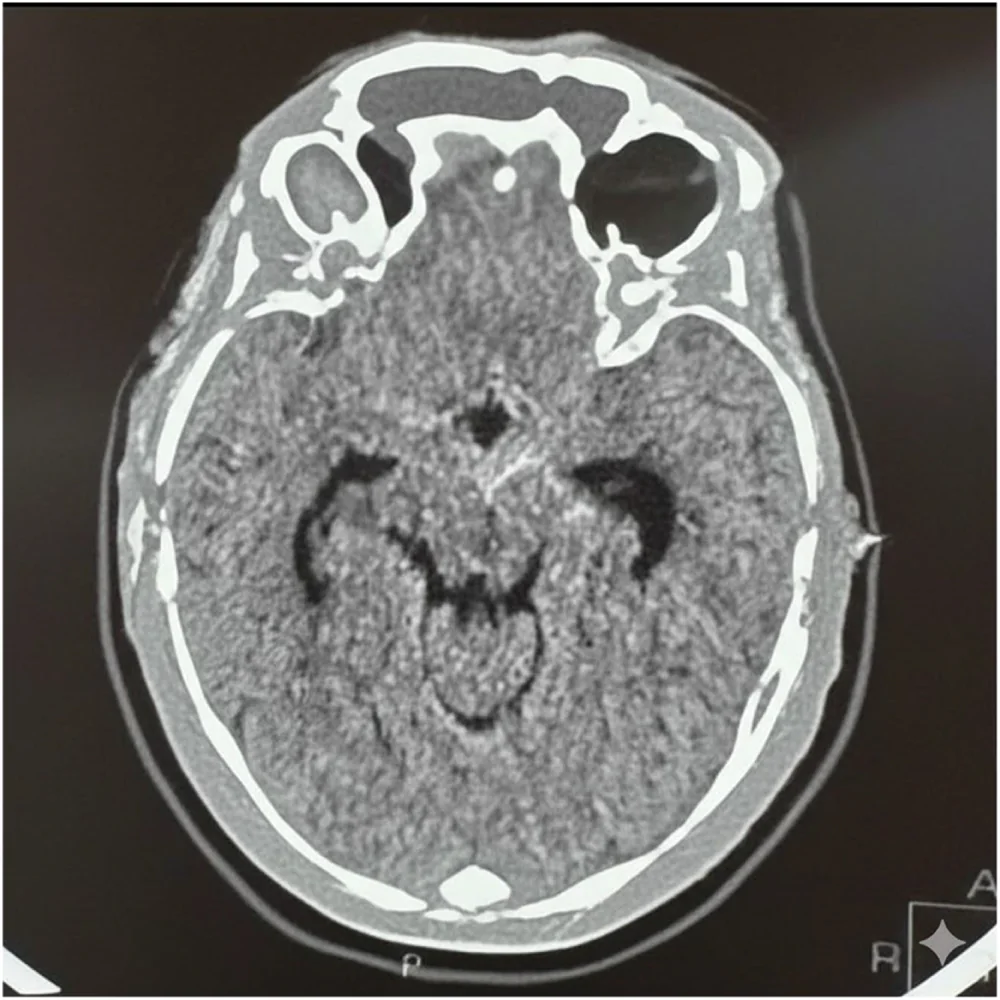
Non-contrast axial CT scan. The imaging shows normal brain parenchyma with preserved sulcal patterns and no acute hemorrhagic or obstructive features. In cases of cerebral venous thrombosis, a native CT is often normal, serving primarily to exclude other acute causes of headache.

On examination, her body temperature was 37.2°C (within the normal range). Neurological examination revealed a fully oriented patient with a Glasgow Coma Scale score of 15. Neck stiffness was present. Cranial nerve examination was normal. Peripheral neurological examination showed normal motor strength and intact sensation. Her blood pressure was 150/90 mmHg, which was high. A non-contrast brain computed tomography (CT) scan was performed, which showed no acute changes (Fig. 1). The blood test results revealed that the D-dimer level was 483 µg/mL (normal range: 0–654 µg/mL), which was within the normal range, suggesting thrombosis was unlikely. Initially, meningitis, stroke, and subarachnoid hemorrhage were considered as possible diagnoses. The patient’s C-reactive protein (CRP) level was 1.89 mg/L (normal range: 0-5 mg/L), and her blood urea nitrogen (BUN) and electrolyte levels were normal.

**Figure 2.**
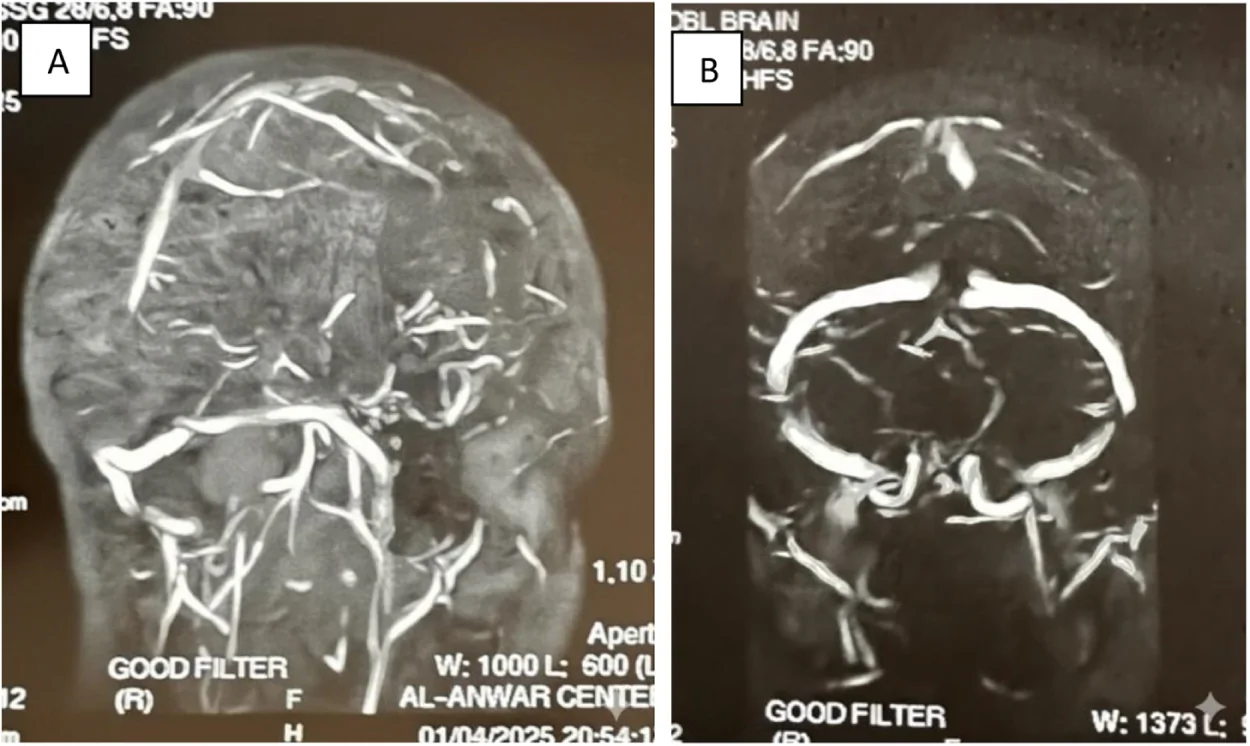
Coronal (A) and transverse (B) contrast-enhanced MR venography (MRV) images of a 49-year-old woman demonstrate intraluminal filling defects within the superior sagittal sinus. These findings are diagnostic of superior sagittal sinus thrombosis and correlate with the patient’s acute neurological presentation.

She was admitted to the internal medicine unit and was initially treated with paracetamol, ondansetron (Zofran), and codeine phosphate for symptomatic relief. When the symptoms persisted and clinical red flags appeared, a contrast-enhanced MRV was ordered. The MRV showed that the superior sagittal sinus was occluded and the flow was interrupted, confirming the diagnosis of SSST. A lacunar infarct was also detected (Fig. 2). Anticoagulation was initiated promptly with intravenous heparin and later switched to oral warfarin. The patient was transferred to the neurology department for further treatment. During the next 11 days, she showed significant clinical improvement and was discharged home on oral anticoagulation (Table 1).

**Table 1:** 

Time point	Event/clinical status
Five days prior to admission	Symptom onset: patient developed a severe, pounding, unilateral (right-sided) headache upon waking. Associated symptoms included photophobia, dizziness, and neck tension.
Day of admission	Clinical presentation: 49-year-old female smoker on oral contraceptives with a history of migraines and hypertension.
	Initial examination: elevated blood pressure (150/90 mmHg) and neck rigidity noted; temperature normal (37.2 °C).
	Preliminary diagnostics: non-contrast CT brain showed no acute changes. Blood tests showed normal D-dimer and normal CRP.
	Initial management: admitted to internal medicine; treated symptomatically with paracetamol, ondansetron, and codeine.
Day 2 of admission	Advanced diagnostics: due to persistent “red flag” symptoms, a contrast-enhanced MRV was performed.
	Diagnosis: MRV confirmed superior sagittal sinus thrombosis with filling defects and a lacunar infarct.
	Intervention: immediate initiation of intravenous heparin; patient transferred to the neurology department.
Days 3–11	Treatment transition: anticoagulation switched from heparin to oral warfarin.
	Clinical progress: gradual improvement in neurological symptoms and headache resolution.
Day 12	Discharge: patient discharged home in stable condition on long-term oral anticoagulation.

## Discussion

### Clinical presentation and red flags

The patient’s symptoms initially appeared to be a severe migraine, which she had experienced for many years. However, several “red flags” suggested a secondary cause. Her headache lasted 5 days, started when she woke up, and included neck stiffness – all unusual for her typical migraine pattern. While a headache is the most common sign of CVST, affecting up to 90% of patients, it does not always feel the same. It can range from a sudden “thunderclap” pain to a chronic, tension-style ache^[^9,23^]^. In this case, the patient’s history of heavy smoking and oral contraceptive use was a major risk factor for blood clots. This combination should lead any clinician to look beyond a simple migraine diagnosis^[^11,12^]^. Additionally, while neck stiffness is a classic sign of meningitis, it can also result from the venous congestion and high brain pressure found in SSST.

### Diagnostic limitations of D-dimer

The normal D-dimer level in this case was a critical finding that could have easily misled clinicians and delayed the diagnosis. While D-dimer is usually a sensitive marker for blood clots, its reliability in detecting CVST is often debated. A meta-analysis by Dentali *et al* found that false negatives are more common in specific scenarios: when a patient has an isolated headache, symptoms lasting over 7 days, or a clot restricted to a single sinus^[^18^]^. Similarly, Kosinski *et al* found that a normal D-dimer level cannot reliably rule out CVST, particularly when a patient already has a high risk of the condition^[^17^]^. Our patient’s profile of 5 days of symptoms and a normal D-dimer level is exactly where the test’s accuracy decreases. This case proves that you should never rely solely on a normal D-dimer to rule out CVST, especially when risk factors and clinical signs suggest otherwise.

### Value of MRV

In this patient, the final diagnosis was confirmed by Magnetic Resonance Venography (MRV); thus, it remains undoubtedly the gold standard for cerebral venous sinus imaging in such cases^[^19,20^]^. A baseline non-contrast CT scan of the head was normal in this patient, and this occurs in nearly 30% of CVST patients^[^23^]^. Regarding CT venography, it might be a quick and effective alternative, showing, for example, the “empty delta sign,” while MRV demonstrates even further details of the thrombus and also of the parenchymal changes adjacent to it, such as the lacunar infarct in this patient^[^21,22^]^. Magnetic resonance imaging/MRV has the capacity to show not only venous occlusion but also its ischemic or hemorrhagic aftermaths; thus, these modalities are a comprehensive source of data that are indispensable for diagnosis and therapy^[^19^]^. This patient’s case illustrates the importance of continuing to advanced venous imaging when there is a strong clinical suspicion of CVST despite a normal non-contrast a scan.

### Therapeutic approach and outcome

After the diagnosis, the patient was promptly started on anticoagulation with heparin and warfarin, which is the mainstay treatment for CVST^[^10,20^]^. Determining factors for treatment with anticoagulants are to stop thrombus propagation, reopen the occluded sinus through recanalization, and prevent new thrombotic events, including deep vein thrombosis and pulmonary embolism. The American Heart Association/American Stroke Association guidelines suggest starting therapeutic anticoagulation even when there is an intracranial hemorrhage, as the benefits of preventing further thrombosis outweigh the risks of hematoma expansion^[^20^]^. The patient’s positive clinical response and the fact that she was released after 11 days reflect the generally good prognosis of CVST if the treatment is initiated without delay. Most patients return to normal function; however, the treatment period (usually 3 to 12 months) is determined by whether the risk factors are transient or permanent^[^10,20^]^. In situations where there is no response to anticoagulation and the condition is severe, it is possible to consider more invasive procedures such as endovascular thrombectomy or thrombolysis^[^24,25^]^.

### Comparison with literature

Our patient’s symptoms align with those described in the general literature on CVST. According to the International Study on Cerebral Vein and Dural Sinus Thrombosis (ISCVT) data, headache was the initial symptom in 89% of cases, and risk factors such as oral contraceptive use were prevalent^[^3^]^. Another commonly reported aspect is the diagnostic journey, which was initially complicated by a misleadingly normal workup. Several studies have consistently reported the variable sensitivity of D-dimer and the low diagnostic yield of non-contrast CT, thus further emphasizing the necessity of having a low threshold for carrying out definitive venous imaging^[^17,23^]^. The patient’s mix of risk factors (female, oral contraceptive use, smoking) makes her part of a high-risk group often referred to in epidemiological studies^[^5,11^]^. On top of that, the favorable response to standard anticoagulation therapy is in line with treatment effectiveness reported in major clinical guidelines and cohort studies that recommend early and intensive anticoagulation as the fundamental therapeutic approach^[^10,20^]^. This report serves as a reminder not to rely too heavily on a single screening test and to follow a diagnostic pathway that is supported by a thorough clinical risk and presentation evaluation.

### Future strategies and ongoing research

Future strategies and ongoing research in the management of SSST primarily aim to refine medical and interventional therapies to improve patient outcomes^[^26^]^. The low incidence of CVST makes large-scale trials challenging^[^26^]^. One major area of focus is the increasing adoption and study of Direct Oral Anticoagulants (DOACs), such as rivaroxaban and dabigatran, for long-term treatment^[^27–29^]^. Studies suggest that these medications are comparable to warfarin in terms of efficacy and recanalization rates, with a potentially lower risk of major hemorrhage^[^27–29^]^. However, large prospective trials are still needed to confirm these findings and update treatment guidelines^[^27–29^]^. For severe or refractory cases, the role of endovascular treatment (EVT), particularly mechanical thrombectomy, is continually evolving^[^30^]^. Research is concentrating on optimizing device selection (e.g., stent retrievers and balloon angioplasty) and defining the best timing for intervention to ensure rapid recanalization while minimizing procedural risks^[^30–32^]^. Current guidelines recommend EVT mainly as a rescue strategy for patients who do not improve despite initial anticoagulation^[^30–32^]^. Additional efforts are also focused on personalizing treatment duration by better integrating individual risk factors, the status of clot recanalization, and genetic thrombophilia markers into clinical decision-making^[^33^]^.

## Conclusion

Our case report outlines an important clinical message, which is that the possibility of CVST must always be considered in patients with unusual or ongoing headaches, particularly those with prothrombotic risk factors. We have shown that a normal D-dimer level should not be considered sufficient to rule out SSST and should not stop further testing if there is ongoing clinical concern. Clinical acumen must always guide the degree of diagnostic testing. For high-risk patients, there should be no delay in obtaining appropriate neuroimaging, especially MRV, to facilitate proper diagnosis. The prompt recognition and initiation of anticoagulation are of utmost importance in management to minimize the risk of significant neurological complications and to ensure that a good outcome is achieved.

## Guidelines

The case has been reported in line with the CAse REport (CARE) guidelines for medical case reports^[^34^]^.

## Data Availability

All data generated or analyzed during this study are included in this published article.

## Research registration unique identifying number (UIN)

Not applicable to this study.
